# Acute effects of a standardised extract of *Hericium erinaceus* (Lion’s Mane mushroom) on cognition and mood in healthy younger adults: a double-blind randomised placebo-controlled study

**DOI:** 10.3389/fnut.2025.1405796

**Published:** 2025-04-10

**Authors:** Geyan Surendran, Jake Saye, Syahira Binti Mohd Jalil, Jack Spreadborough, Kyle Duong, Israa M. Shatwan, Dash Lilley, Michael Heinrich, Georgina F. Dodd, Shelini Surendran

**Affiliations:** ^1^Beyond Alcohol Ltd., London, United Kingdom; ^2^Faculty of Health and Medical Sciences, University of Surrey, Guildford, United Kingdom; ^3^Food and Nutrition Department, Human Sciences and Design Faculty, King Abdulaziz University, Jeddah, Saudi Arabia; ^4^Pharmacognosy and Phytotherapy, UCL School of Pharmacy, London, United Kingdom; ^5^Graduate Institute of Integrated Medicine, College of Chinese Medicine, Chinese Medicine Research Center, China Medical University, Taichung, Taiwan; ^6^Clasado Ltd, Imperium Building, Imperial Way, Worton Grange, Reading, Berkshire, United Kingdom

**Keywords:** mushroom, Lion’s Mane mushroom, *Hericium erinaceus*, cognition, mood

## Abstract

**Introduction:**

Animal studies have suggested that Lion’s Mane mushroom [*Hericium erinaceus* (Bull.) Pers.] can enhance cognitive function and mood due to its bioactive metabolites, including erinacines and hericenones. However, despite being an ingredient used both culinarily and therapeutically in the East, and more and more commonly in the West, limited research has focused on the immediate effects of *H. erinaceus* on the cognitive function and mood of healthy young adults.

**Methods:**

In an acute randomized, placebo-controlled, double-blinded, cross-over intervention study, we investigated the potential benefits of an acute dose of *H. erinaceus* fruiting body extract (3g of 10:1 extract) on cognitive performance and mood compared to a placebo. Eighteen healthy participants aged 18 to 35 years took part in the study. At baseline and 90 minutes post-consumption of the interventions, cognitive and mood assessments were administered to measure various cognitive abilities such as executive function, working memory, psychomotor skills, attention and information processing speed as well as positive and negative affect.

**Results and discussion:**

The results showed no significant effect of the *H. erinaceus* fruiting body extract for composite measures of global cognitive function and mood. However, when analysing individual tests, participants exhibited improved performance on the pegboard test at 90 minutes following a single dose of *H. erinaceus*.

**Conclusion:**

In conclusion, acute consumption of *H. erinaceus* fruiting body did not demonstrate a significant overall improvement in cognitive performance and mood compared to the placebo and any benefits may be task or domain specific. Further investigations should investigate the effects of chronic supplementation of *H. erinaceus* fruiting body on cognition and mood in healthy younger adults, as well as establish optimal dosage and the time to peak concentration of *H. erinaceus* bioactives in the human brain. Additionally, future research should aim to further elucidate potential mechanisms of action to explain potential brain region and cognitive domain specific effects, such as possible regional increases in cerebral blood flow following consumption of *H. erinaceus* fruiting bodies. It must also be noted that typically only Lion’s mane fruiting bodies are consumed culinarily, where up to 300g of fresh fruiting body are often consumed in the form of mushroom steaks.

## Introduction

*Hericium erinaceus* (Bull.) Pers. (Hericiaceae; Engl,: Lion’s Mane mushroom), is a toothed fungus endemic to the Northern Hemisphere. This unique looking fungus has garnered significant attention in recent years for its potential cognitive and mood-enhancing properties. Traditionally used in East Asian cuisine and medicine, this distinctive fungus has seen a surge in popularity as both a culinary ingredient and dietary supplement in Western countries.

While much research has focused on the effects of *H. erinaceus* in populations with cognitive impairment or mood disorders, there is growing interest in its potential benefits for healthy individuals. Preclinical studies have demonstrated that bioactive compounds in lion’s mane, such as hericenones and erinacines, can promote nerve growth factor (NGF) synthesis and potentially support neuroplasticity (1). These findings suggest that consuming Lion’s mane may have cognitive benefits even in the absence of pathology.

Addressing cognitive and mood impairments is increasingly important for public health. Neurological diseases contribute significantly to physical and cognitive decline, affecting 15% of the global population (2). The prevalence of mood disorders, such as anxiety and depression, is also rising. In 2019, anxiety disorders affected 4.05% of the global population, a 55% increase from 1990, with rates rising during the COVID-19 pandemic (3, 4).

Due to the increasing prevalence of neurodegenerative and mood disorders, it is more vital than ever to discover treatments that may help to alleviate these ailments. *Hericium erinaceus* has shown potential in treating a wide range of Central Nervous System (CNS) disorders. *H. erinaceus* contains a range of different compounds with a variety of biological activities (1) including erinacines and hericenones, which enhance growth factor (NGF) release (5).

Tsai-Teng et al. (6) demonstrated that erinacine A-enriched *H. erinaceus* supplementation significantly attenuated amyloid *β* plaque deposition in transgenic mice, indicating potential therapeutic effects for Alzheimer’s disease. Additionally, *H. erinaceus* extracts have shown antidepressant-like effects in mice, acting through mechanisms such as hippocampal neurogenesis (7) and induction of the brain-derived neurotrophic factor (BDNF) pathway, which is critical for neuronal survival and neurogenesis (8). Another murine study found that *H. erinaceus* increased levels of serotonin, dopamine, and noradrenaline, neurotransmitters that are often decreased in depression (9).

Human studies on *H. erinaceus* and depression are limited. One study by Vigna et al. (10) found that 8 weeks of *H. erinaceus* supplementation improved anxiety and depression measures in overweight or obese participants, also increasing circulating pro-BDNF levels. *H. erinaceus* has also shown potential in improving cognitive functions in individuals with mild cognitive impairment. Mori et al. (11) found cognitive improvement after 16 weeks of 250 mg daily *H. erinaceus* supplementation in older adults. Saitsu et al. (12) demonstrated cognitive improvements in participants consuming 2.4 g of *H. erinaceus* daily for 12 weeks. Docherty et al. (13) found an increase in performance on the Stroop task (a cognitive test measuring response inhibition) following a single 1.8 g dose of *H. erinaceus* in a young, healthy population indicating acute cognitive-benefits of *H. erinaceus*. This study also indicated an improvement in reported stress following 28-days chronic supplementation with *H. erinaceus*.

Our study extends recent research by Docherty et al. (13) by focusing solely on the immediate effects of a higher, more concentrated dose derived exclusively from the fruiting body of *H. erinaceus*. This approach addresses the fact that within the EU and UK, mycelial extracts or preparations of lion’s mane mycelia are not allowed to be consumed within food products or nutritional supplements however fruiting body extracts are permitted (14). The distinction between fruiting body and mycelial extracts is significant due to the differing profiles of bioactive compounds. Fruiting bodies are rich in hericenones, which have been shown to enhance cognitive function and mood (1). In contrast, mycelia contain higher concentrations of erinacines, known for their neurotrophic effects.

The effects of *Hericium erinaceus* on cognition (15, 16) and mood have received considerable attention, with research primarily focusing on its benefits for individuals with nervous system diseases. However, the potential cognitive and mood benefits of *H. erinaceus* in healthy individuals are also of significant interest. Our study investigates these acute effects in a young, healthy population, which is a less commonly studied demographic. By using an extract derived specifically from the fruiting body of *H. erinaceus,* our research aims to provide unique insights into its efficacy for cognitive enhancement in healthy adults.

## Methodology

### Participants

Participants eligible for inclusion in the trial were healthy adults aged between 18 and 35 years and located in the United Kingdom. The exclusion criteria were as follows: pregnant or lactating women, those concurrently participating in any other clinical trial within 6 weeks prior to the commencement of the current trial, individuals who had made significant changes to their diet or lifestyle in the previous month, those with a Body Mass Index (BMI) of 30 or above, individuals with psychiatric or medical conditions, those with clinician diagnosed depression, individuals with existing neurological impairments or diseases, individuals with existing cardiovascular disease or complications, individuals with a previous history of diabetes, individuals taking medication with stimulatory or sedative effects, and those on regular medication for heart disease, hypertension, liver or kidney disease, high cholesterol, autoimmune disease, cancer, psychiatric disorders, or diabetes. Additionally, individuals who were currently smoking or addicted to substances, regularly consuming functional foods (commonly defined foods consumed to be beneficial to health beyond basic nutrition) or dietary supplements with cognitive-enhancing effects similar to *H. erinaceus* (or unwilling to stop during the study), those with food allergies or intolerances, individuals engaging in frequent vigorous exercise, those with learning difficulties (e.g., dyslexia), asthma, hypoglycemia, bleeding disorders, or those unable to provide consent for themselves were also excluded from participation in the trial.

The software package G*power (17) was used to determine sample size *a priori*. Based on an effect size f of 0.3 [which was expected according to the existing literature – see (13)], an alpha of 0.05 and power of 80%, employing a cross-over design and planning to perform the associated within-subject F test for the primary endpoint, the G*Power program determined that a sample size of 17 would be acceptable. Furthermore, as noted by Docherty et al. (13), for the purposes of a pilot study, a sample size of between 12 and 30 per group is recommended (18, 19), therefore, considering potential drop-outs, the aim was to recruit 20 participants in total.

### Interventions

The *H. erinaceus* extract (95% *H. erinaceus* extract and 5% maltodextrin; Cambridge Commodities Ltd.) was derived from its fruiting body through a process of water and ethanol extraction (8:2), followed by spray drying over a maltodextrin substrate. The country of origin and manufacture of the *H. erinaceus* extract was China. The decision to use fruiting body extracts was based on the prohibition of using mycelial extracts within the European Union within EU novel foods guidelines, as well as its established use within commercially available, over the counter supplements and food products.

It must be acknowledged that there are some significant differences between the profiles of bioactive compounds between the fruiting bodies and mycelia, which could in turn influence their physiological activity.

Extraction methods can also have a marked impact on the efficacy of an extract. This study employed both aqueous and ethanolic extraction methods, which are widely used for their ability to extract both polar and non-polar compounds. These methods maximise the yield of bioactive compounds without relying on non-potable solvents, thus balancing efficiency and consumer perceptions of safety. The resulting extract is then spray dried, allowing the concentration of bioactives and prevention of degradation by environmental conditions or microbes (20), while ensuring that they are evenly distributed across an easily storable, transportable medium that is convenient to handle and measure prior to further processing or final consumption.

The 250 mL *H. erinaceus* drink (active drink) was comprised of 3 g of the *H. erinaceus* extract mixed with 220 mL of water and 30 mL of Robinson’s Lemon Squash as a vehicle. The control drink (control drink) was matched to the active drink in terms of volume, appearance, taste, and macronutrient composition. The composition of the *H. erinaceus* and control drinks is provided in Tables 1, 2.

**Table 1:** Composition of the *H. erinaceus* extract and control drinks.

	Control drink	*H. erinaceus* extract drink
*Hericium erinaceus extract* (g)	0	3
Lemon Squash (mL)	30	30
Water (mL)	220	220

**Table 2:** The nutritional content of *H. erinaceus* extract and control drinks.

Nutrients	Control drink**	*H. erinaceus* drink*
Calories (kcal)	3	4.29
Fats (g)	0	0
Carbohydrates (g)	0	0.2
Fibre (g)	0	0.06
Sugar (g)	0	0.06
Salt (g)	0.10	0.10
Protein (g)	0	0.06
Sodium (mg)	0	0.3
Potassium (mg)	0	9.2
Calcium (mg)	0	0.06
Iron (mg)	0	0.01

^*^Amount per 3 g of *H. erinaceus* extract diluted in 250 mL of vehicle (30 mL lemon squash and 220 mL of water).

^**^Amount in 250 mL of vehicle (30 mL lemon squash and 220 mL of water).

### Study design

**Figure 1:**
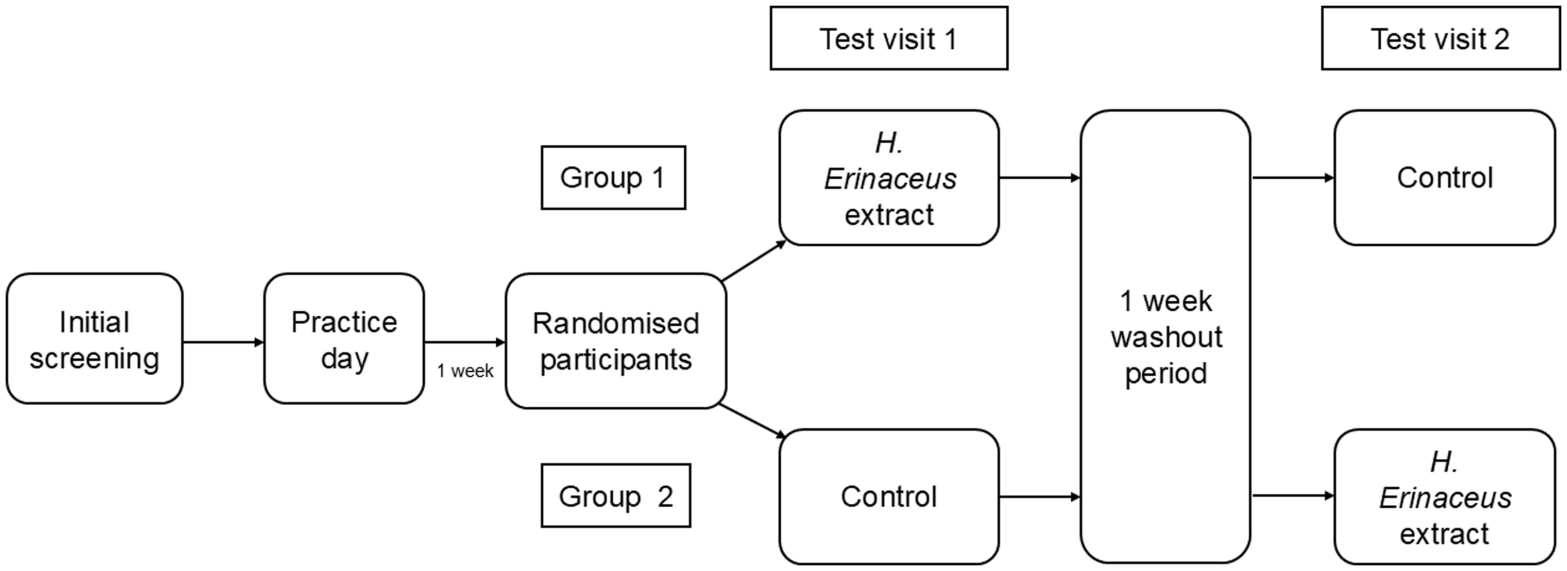
Study design.

Participants consumed both the *H. erinaceus* extract drink (active drink) or the control drink (control drink) in accordance with a double-blinded, placebo-controlled, acute, cross-over study design (Figure 1), with the order of consumption counterbalanced across the sample and a seven-day washout between test days. Cognitive function and mood were assessed at baseline (pre-consumption) and at 90 min post-consumption of the test beverages (see procedure). A familiarisation/practise day occurred 7 days prior to the first test day, during which no drink was consumed. Participants were then randomised into two groups to determine whether they would consume the active drink or control drink first, using computer-based non-deterministic randomisation in Microsoft Excel. Group 1 consumed the *H. erinaceus* extract on test day 1 and control on test day 2 whereas for group 2, the order of consumption of the test beverages was reversed.

### Procedure

Upon recruitment and after obtaining informed consent, participants were invited to attend a baseline familiarisation/practise day during which demographic information was collected, eligibility for participation in the trial was confirmed, and a practise version of the cognitive battery was administered. The purpose of familiarising the participants with the cognitive tests prior to the first test day was to reduce the disproportionate number of errors during the initial testing session, the ultimate aim of which was to minimise any practise effects. Researchers were present throughout the practise session to answer any questions participants might have about the tasks and ensure they understood the instructions.

For the 24 h prior to each visit, participants followed a restricted diet which included the prohibition of alcohol consumption. Participants also fasted for the 12 h prior to the visit. These measures were taken in order to prevent any potential acute effects of foods/beverages that could influence cognitive performance, such as caffeine (21). Extreme exercise (for example weightlifting and high intensity interval training) were also prohibited for the 24 h prior to each visit, as exercise has also been shown to alter cognitive function (22) and extreme exercise can potentially introduce confounding factors, such as fatigue which could affect test performance. All test visits took place at the clinical investigation unit (CIU) of the University of Surrey in March 2023 at 9 am. During test days, participants were given 15 min to consume a standardised breakfast of a croissant and water upon arrival. The participants then carried out a series of baseline measurements of cognition and mood which takes approximately 1 h to complete the tasks, depending on the individuals’ speed.

Following the baseline test session, the participants were asked to consume either the active drink or control drink within 15 min, depending on which intervention they had been randomly assigned to consume at that test visit. Half an hour before consumption, a confederate prepared the drinks, which were consumed with a black straw and a vessel that obscured the beverage from the subject, ensuring double-blinding. The confederate observed the subject throughout to ensure the drink was consumed in its entirety. After a delay of 90 min the same cognitive and mood assessments were repeated.

On completion of the initial test day, participants observed a washout period of one-week. Following this, they attended the clinic for a second test day during which the same procedure as for the first was repeated, but participants were given the drink they did not consume on their first test visit. Participants were given parallel versions of the cognitive tasks at the different testing timepoints to minimise learning effects.

### Outcomes

The primary outcome was global cognitive function as determined via a composite measure reflecting combined performance across all of the cognitive tasks administered. The cognitive test battery included the following tasks: Trail Making Test (TMT) parts A and B (27, 48), Digit Span Test (DST) (29, 45), Digit Symbol Substitution Test (DSST) (28, 46), Grooved Pegboard Test (GPT) (47), Deary-Liewald Task (23), and the Flanker Task (25, 44). The TMT is commonly used to measure executive function, the DST to measure working memory, the DSST is a measure of information processing, the GPT psychomotor skills, whilst the Deary-Liewald task measures reaction time and processing speed, and finally the Flanker task is a measure of inhibitory control which is often classed as an aspect of executive function (24). The references provided for the cognitive tests in this methods section are intended to provide further details of each and explain how they are administered in practice, to aid replicability. Dependent variables were either accuracy or reaction time as appropriate for the task.

Secondary outcomes were overall mood (composite measure) as measured by the Positive and Negative Affect Schedule (PANAS) (26), performance on each individual cognitive task and positive and negative affect separately. The PANAS is a psychometric questionnaire comprised of 20 mood-related adjectives (e.g., ‘hostile’, ‘alert’, ‘afraid’, ‘excited’) for which the participant has to indicate on a scale of 1–5 with 1 being ‘very slightly/not at all’ and 5 being ‘extremely’. Typically this questionnaire is used to determine how often they have felt that way over the past week, however it can also be used to determine acute changes to mood by asking volunteers to answer the questions based on how they currently feel.

The study’s cognitive and mood test battery included both paper-based tasks (Trail Making Test, Digit Symbol Substitution Test, and Digit Span Test) as well as computerised tasks hosted on Gorilla.sc (Deary-Liewald Task, Flanker Task, and PANAS questionnaire), benefiting from its ability to be robust to environmental variance (30). The grooved pegboard test, separate from the paper and computerised tasks, measured motor dexterity by scoring the number of pegs placed into a board within 30 s using both preferred and non-preferred hands. These tests were completed at baseline and 90 min post-intervention, with parallel versions of the cognitive tasks administered at both timepoints.

### Ethical approval

This study was granted ethical approval by the University of Surrey Research Integrity & Governance Office (RIGO), FHMS 22-23 117 EGA. This study was conducted in compliance with the Declaration of Helsinki, relevant University policies, ethical and professional standards, and all mandatory training was provided by the University of Surrey.

### Statistical analysis

Raw scores were standardised by converting to Z scores. The grand means (*X*) and standard deviations (*SD*) across all conditions and timepoints for each of the cognitive tests were determined. Z scores were then calculated by subtracting the grand mean of the relevant cognitive test from each raw score then dividing by the *SD* of that test [i.e., (*μ* – X)/*SD* where μ is the raw score]. For Deary Liewald Task, Flanker test and PANAS negative outcomes, Z scores were inverted in order that for all variables, a higher score indicated better cognition or mood (the inversion was achieved by multiplying the relevant Z scores by −1). Calculating Z scores overcomes the issue of differences in the nature of dependent variables (e.g., units of measurement) across the various cognitive tests when combining data into composite measures.

A global composite measure of overall cognitive performance (primary endpoint) was calculated for every subject for each condition and timepoint. This was achieved by summing the Z scores across the different cognitive tests and dividing by the number of cognitive tests contributing to the composite measure. A composite measure of mood was calculated using the same method. It is important to calculate composite measures of cognition for comparability with other studies (31, 32).

Considering the cross-over design of the study, with measurements at baseline and post-consumption of the drinks, such that all subjects completed both drink conditions and measurements at each of the timepoints, the global composite measures of cognition and mood were analysed using a two-factor (Intervention; Time) repeated measures analysis of variance (ANOVA) with Intervention (active drink; control drink) and Time (Baseline; Post-drink) as within-subject factors.

Z score data for the individual cognitive and mood measures were analysed using a two-factor (Intervention; Time) repeated measures multivariate analysis of variance (MANOVA), which enables the effect of independent variables and their interaction on several dependent variables to be analysed in one analysis, allowing for adequate control over type 1 error.

For all statistical analyses, Bonferroni corrected pairwise comparisons were examined for Treatment related effects regardless of the significance of the overall multi-or univariate test statistic [see (33)] and a significance level of *p* < .05 was applied.

IBM SPSS statistics version 29.0.0.0 was used to conduct all statistical analyses.

## Results

**Figure 2:**
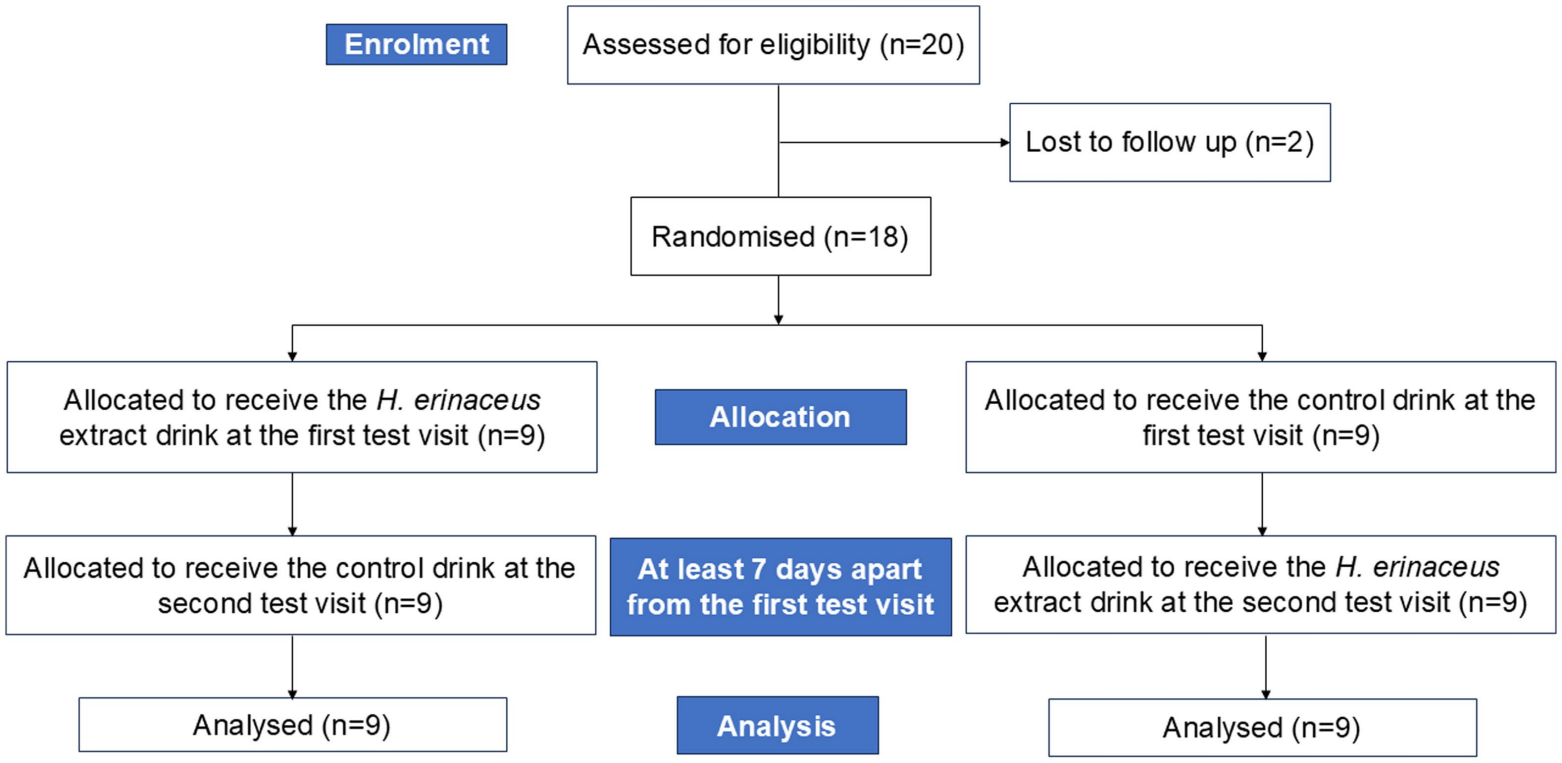
Consort diagram showing group assignment.

A total of twenty adults were initially recruited, however two participants failed to attend the familiarisation/practise day, therefore eighteen participants of which 10 were male and 8 were female (see Table 3 for demographic information of the sample) were randomised and all completed the study (see Figure 2). Only main effects and interactions related to Intervention are reported as these are the most pertinent to establishing the acute effects of *H. erinaceus* on cognition.

**Table 3:** Demographic information of participants.

	*M*	*SD*	Range
Age (years)	22.78	4.09	18–35
BDI^1^ score (/63)	8.47	5.47	0–19
Height (cm)	168.83	7.71	158.8–185
Weight (kg)	61.72	9.04	45.5–85

^1BDI, Beck Depression Inventory.^

There was no significant main effect of Intervention (active drink vs. control drink), or an Intervention x Time interaction for the composite measure of global cognitive function (all *p* > 0.05) and the composite measure of mood (all *p* > 0.05), see Table 4 for means and standard deviations. The higher the value, the better the cognitive performance or mood.

**Table 4:** Z scores for the global cognitive and mood composite measures for each condition and timepoint.

	Control - Baseline		Control - Post-drink		*H. erinaceus* - Baseline		*H. erinaceus* - Post-drink	
	*M*	*SD*	*M*	*SD*	*M*	*SD*	*M*	*SD*
Global cognition composite measure^1^	0.001	0.50	0.01	0.26	−0.07	0.39	−0.04	0.48
Mood composite measure^1^	0.02	0.58	−0.06	0.65	−0.06	0.74	0.10	0.64

^1See the results section as to how these were calculated.^

However, the MANOVA of the individual cognitive and mood measures revealed a main effect of Intervention (*V* = 0.81, *F* (10.8) = 3.30, *p* = 0.05, η_p_^2^ = 0.81) as well as an Intervention x Time interaction (*V* = 0.79, *F* (10.8) = 2.94, *p* = 0.07, η_p_^2^ = 0.79) that approached significance.

In relation to the trend for a main effect of Intervention, examination of the Univariate tests suggest this could be due to effects for the Pegboard – Dominant (*F* (1.17) = 10.41, *p* = 0.01, η_p_^2^ = 0.38) and Flanker (*F* (1.17) = 4.50, *p* = 0.049, η_p_^2^ = 0.21) tasks, with pairwise comparisons showing better performance in the active drink compared with the control drink condition for the Pegboard - Dominant task (mean difference = −0.40, *SE* = 0.12, *p* = 0.01; active drink *M* = 0.20, *SE* = 0.23; control drink *M* = −0.20, *SE* = 0.19) but a trend for worse performance in the active drink condition compared to control drink for the Flanker task (mean difference = 0.38, *SE* = 0.18, *p* = 0.05; active drink *M* = −0.19, *SE* = 0.13; control drink *M* = 0.19, *SE* = 0.21).

However, perhaps of most interest and relevance is the trend toward an Intervention x Time interaction, with Univariate tests revealing this could be due to effects for the Pegboard – Dominant (*F* (1.17) = 15.75, *p* < 0.001, η_p_^2^ = 0.48), Pegboard – Non-dominant (*F* (1.17) = 19.31, *p* < 0.001, η_p_^2^ = 0.53), and Flanker (*F* (1.17) = 4.87, *p* = 0.04, η_p_^2^ = 0.22) cognitive tasks and possibly the Trails B (*F* (1.17) = 4.42, *p* = 0.05, η_p_^2^ = 0.21) task and PANAS-positive mood measure (*F* (1.17) = 3.59, *p* = 0.08, η_p_^2^ = 0.17), although notably the effects for the latter two measures were only trends.

**Figure 3:**
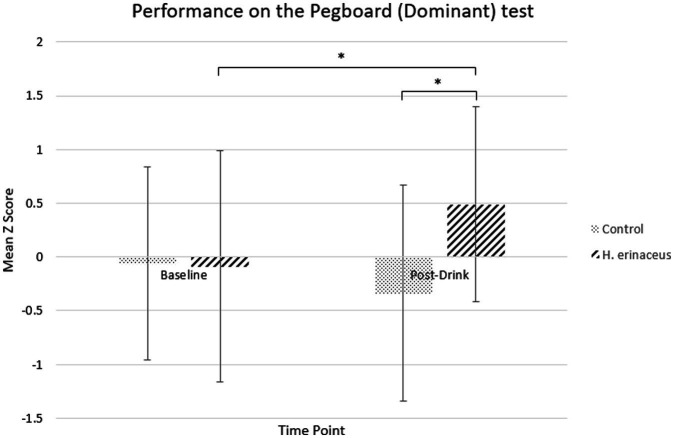
Performance on the pegboard (dominant) test for both interventions (*H. erinaceus* and control) at each timepoint.

Pairwise comparisons showed significantly better performance in the active drink condition compared to control drink at the post-drink timepoint for the Pegboard – Dominant task (mean difference = −0.83, *SE* = 0.14, *p* < 0.001; active drink *M* = 0.49, *SE* =0.21; control drink *M* = −0.34, *SE* = 0.24) with no significant difference at baseline (*p* > 0.05). Furthermore, there was significant improvement in performance following the active drink compared to baseline (mean difference = −0.58, *SE* = 0.12, *p* < 0.001; Baseline *M* = −0.09, *SE* = 0.25; Post-drink *M* = 0.49, *SE* = 0.21), which was not observed for the control drink (Figure 3).

For the Pegboard – Non-dominant task, although performance was significantly better in the control drink compared with the active drink condition at baseline (mean difference = 0.72, *SE* = 0.28, *p* = 0.02; active drink *M* = −0.35, *SE* = 0.20; control drink *M* = 0.37, *SE* = 0.31), performance significantly increased following the active drink compared to baseline for this task (mean difference = −0.52, *SE* = 0.12, *p* < 0.001; Baseline *M* = −0.35, *SE* = 0.20; Post-drink *M* = 0.17, *SE* = 0.23), whereas there was a significant decline in performance following the control drink (mean difference = 0.55, *SE* = 0.23, *p* = 0.03; Baseline *M* = 0.37, *SE* = 0.31; Post-drink *M* = −0.18, *SE* = 0.16).

Furthermore, for the Trails A task, performance significantly decreased following the control drink compared to baseline (mean difference = 0.35, *SE* = 0.12, *p* = 0.01; Baseline *M* = 0.11, *SE* = 0.22; Post-drink *M* = −0.24, *SE* = 0.16).

Conversely, for the Flanker task, performance was significantly worse for the active drink condition compared with control drink (mean difference = 0.84, *SE* = 0.20, *p* < 0.001; active drink *M* = −0.37, *SE* = 0.16; control drink *M* = 0.47, *SE* = 0.09) at the post-drink timepoint (no significant difference at baseline, *p* > 0.05).

Also, for the Trails B task, a significant decline in performance was evident following the active drink compared to baseline (mean difference = 0.42, *SE* = 0.13, *p* = 0.01; Baseline *M* = 0.13, *SE* = 0.20; Post-drink *M* = −0.28, *SE* = 0.21).

There were no other significant main effects, interactions or pairwise comparisons.

Z score data (means and standard deviations), for each cognitive and mood measure are shown in Table 5. The higher the value, the better the cognitive performance or mood.

**Table 5:** Z scores for each of the cognitive and mood measures for each condition and timepoint.

	Control - Baseline		Control - Post-drink		*H. erinaceus* - Baseline		*H. erinaceus* - Post-drink	
Measure	*M*	*SD*	*M*	*SD*	*M*	*SD*	*M*	*SD*
TMT^1^ - part A	0.11	0.91	−0.24	0.66	0.42	1.30	−1.00	3.38
TMT^1^ - part B	0.06	0.94	0.09	1.31	0.13	0.84	−0.28	0.87
Digit Span	0.07	1.14	0.05	1.02	−0.21	0.82	0.10	1.04
DSST^1^	−0.39	1.02	0.12	0.82	−0.25	1.04	0.51	0.93
Pegboard - Dominant	−0.06	0.90	−0.34	1.00	−0.09	1.08	0.49	0.91
Pegboard - Non-dominant	0.37	1.32	−0.18	0.66	−0.35	0.83	0.17	0.99
Deary-Liewald Task	−0.06	0.84	0.12	0.86	−0.17	0.92	0.10	1.35
Flanker Task	−0.09	1.63	0.47	0.37	−0.01	0.71	−0.37	0.69
PANAS^1^ - Negative	−0.05	1.01	−0.03	1.03	−0.06	1.15	0.15	0.87
PANAS^1^ - Positive	0.09	1.01	−0.08	1.07	−0.06	1.01	0.05	0.98

^1TMT, Trail Making Test; DSST, Digit Symbol Substitution Test; PANAS, Positive and Negative Affect Schedule.^

## Discussion

This is one of the few studies to investigate the acute effects of *H. erinaceus* extracts on cognition and mood in healthy younger adults. Whilst there was no overall effect on cognition as determined by the global composite measure, when cognitive tasks were analysed individually, acute consumption of *H. erinaceus* resulted in improved psychomotor skill. However, there was no evidence for a beneficial effect of the extract on mood in this study.

Interestingly, our investigation unveils the potential advantages of a single *H. erinaceus* dose on psychomotor skill/manual dexterity in a young and healthy cohort, as evidenced by improved performance on the pegboard test (dominant and non-dominant forms). The pegboard task is a widely used measure of psychomotor performance and manual dexterity. In this task, participants are required to insert pegs into holes on a board as quickly and accurately as possible. It assesses hand-eye coordination, fine motor skills, and the speed of information processing. The primary outcome measure for this task is the total number of pegs correctly placed within a specified time limit. Including the pegboard task in our study aimed to capture any potential acute effects of *H. erinaceus* on these specific cognitive and motor abilities.

The nuanced nature of acute cognitive effects seen with *H. erinaceus* extract in the current trial are in agreement with those of a recent trial in a similar population by Docherty et al. (13) who also observed an acute benefit to cognition only for a specific test measuring a particular cognitive ability. However, whilst in this acute trial we did not observe any beneficial effects on mood, Docherty et al. (13) reported a trend toward reduced subjective stress following chronic supplementation, suggesting that whilst specific cognitive effects can occur acutely in healthy younger adults, mood effects may only be evident following chronic supplementation.

Conversely, acute consumption of the *H. erinaceus* extract appeared to impair performance on the Flanker and Trail making B tests, both of which assess aspects of executive function (34, 35). Taken together, these conflicting results suggest that the effects of *H. erinaceus* extract on cognitive function in a young population 90 min after a single (3 g) dose are inconclusive based on this study. The lack of conclusive evidence on the beneficial cognitive effects of *H. erinaceus* extract is in alignment with the findings of a study conducted by Grozier et al. (36), who also measured cognitive changes in a young, healthy adult population after 4 weeks of *H. erinaceus* extract consumption and found no significant changes in cognition. However, considering this was a trial with a relatively small sample size, all cognitive results should be interpreted with caution and require replication in future studies to validate the findings. Furthermore, the mechanisms that underlie potential acute cognitive benefits of *H. erinaceus* are largely unknown and establishing these could be an aim of future research.

Previous studies demonstrating positive effects of *H. erinaceus* on depression and anxiety, such as those by Okamura et al. (37) and Vigna et al. (10), took place over 4 and 8 weeks, respectively. In the 2019 study by Vigna et al., participants were supplemented with a 500-milligram capsule, using an 80:20 mycelium to fruiting body ratio with each individual ingesting 84 grams of *H. erinaceus* over an eight-week period. These findings may be attributed to its role in impacting monoaminergic pathways, promoting neurogenesis and neurotrophic factors expression (including nerve growth factor), and/or exhibiting anti-inflammatory effects by modulating expression levels of IL-6, TNF-*α*, and NF-κB (38). Our acute study showed no statistically significant improvements in positive and negative mood as measured by the PANAS after *H. erinaceus* consumption when compared to the control. Our findings align with those of a study in a population of healthy adults where a single dose of *H. erinaceus* did not result in a reduction in stress. However, when supplemented for 28-days, a trend toward reduced subjective stress was observed (13). Most previous studies were conducted on populations that were depressed, whereas our study only included individuals without any clinician diagnosed mood disorders. It may be the case that *H. erinaceus* only confers positive mood changes in those experiencing mood disorders. Another possibility is that there is less measurable variance post-intervention in those with stable mood patterns such as the population we tested in this study.

A notable aspect of this study is that neither the *H. erinaceus* extract beverage nor the control contained additional active ingredients. Thus, any cognitive improvements are likely due to *H. erinaceus* metabolites. The study used a dosage of 3 grams of extract, equivalent to 30 grams of fresh lion’s mane fruiting body, which is higher than previous trials that typically used lower dosages. Furthermore, this study focused solely on the fruiting body, while culinary consumption of lion’s mane can be tenfold this amount. The 5% maltodextrin content, incidental from the spray drying process, is unlikely to significantly affect cognitive tests. It is possible the positive changes in mood and cognition that occurred in previous studies take longer than 90 min or are only evident following chronic supplementation. Due to the difficulty in observing measurable change in healthy individuals compared to those with cognitive decline, should benefits in such a population be evident, a longer period of supplementation may be necessary in order to observe them.

Future research might benefit from encapsulating the extract, as olfactory stimuli can influence mood and cognition (39). Positive changes in mood and cognition might take longer than 90 min to manifest or require chronic supplementation. Observing measurable changes in healthy individuals can be challenging; hence, a longer supplementation period may be necessary to detect benefits.

As this was an investigation with 18 participants, larger randomised controlled trials with a wide range of doses are warranted in order to demonstrate the potential beneficial effects of a single dose of *H. erinaceus* on mood and cognition. In addition, when designing acute trials, researchers could also consider increasing the delay between consumption of the test interventions and the post-intervention cognitive test session to more than 8 h. Previous studies have indicated that erinacine S, thought to be one of the bioactive compounds in *H. erinaceus,* can be detected in rats 2 h after administration, reaching peak concentration 8 h after oral administration in rats (40). However, this was not considered to be practical for the present human intervention study and due to the limited research of *H. erinaceus* in humans, the time it takes for erinacine S to reach peak concentration in human plasma is currently unknown, therefore post-intervention testing after a delay of 90 min was considered appropriate for this study. Furthermore, it is important to note that different organisms have different metabolism rates, therefore a study to determine bioavailability in humans would be warranted. It may also be prudent to test at more than one post-intervention timepoint to increase the likelihood of detecting any cognitive and mood effects and to determine changes over time.

Lion’s Mane mushroom (LMM) extracts may yield varying benefits due to differences in active ingredients. Not all LMM extracts contain equal proportions of purported bioactives. Variability arises from factors such as the source of raw material, extraction methods, and extract constituents. For example, extracts derived from the fruiting body of *Hericium erinaceus*, as regulated by the EU Novel Foods Catalogue (14), differ from those utilising both fruiting body and mycelium. These differences are underscored by research showing distinct bioactive profiles between the fruiting body and mycelium (1, 41). Moreover, the use of solvents such as ethanol, methanol, or water significantly impacts bioactive yield and consumer acceptance. While water extraction aligns with preferences for natural and safe products, ethanol extraction is more favoured in extraction of terpenoids including hericenones, as highlighted by Chang et al. (42).

Aqueous extractions have been used traditionally in foods, extensively, such as in soups and mushroom teas made from the fruiting body, remain traditional and widely accepted, whereas using organic solvents or supercritical CO2 extraction methods (43) —which may enhance yield and stability— require more consumer awareness. Historically tinctures using mushrooms as a traditional medical product were extracted by ethanol, but there is no evidence it has been used for food. Additionally, techniques like spray drying improve extract stability and ease of use (20), addressing consumer and regulatory expectations.

While this study utilised a *H. erinaceus* extract derived from the fruiting body, future research could delve deeper into analysing factors affecting efficacy. This highlights the importance of considering *Hericium erinaceus* extract variability in future studies. Future research should also consider including clinical and biochemical parameters, as well as quantifying bioactives within the administrative extract to help elucidate the potential mechanisms of action behind any beneficial effects of *H. erinaceus* on cognition and mood.

In conclusion, this study investigating the effect of *H. erinaceus* on cognition and mood in healthy younger adults was inconclusive. Whilst improved scores on certain cognitive measures suggest potential benefits to motor dexterity, this finding was contradicted by negative results in other tests. Furthermore, no beneficial effects on mood were detected, possibly due to the research being conducted on healthy participants, the dosage of *H. erinaceus* administered and the acute design of the study with only a single dose consumed. To date there are still a limited number of studies looking at the effects of *H. erinaceus* in humans. Future studies looking at the acute effects after a single dose of *H. erinaceus* should consider appropriate dosing for the population of interest, as well as the time between consumption and testing, or potentially testing at several time points following consumption. Further studies in healthy populations could clarify the mixed results of this study and expand our knowledge of the therapeutic potential of *H. erinaceus* for the wider population. It would also be interesting to investigate acute supplementation with *H. erinaceus* in compromised populations where there may be a greater opportunity to observe cognitive and mood benefits. Finally, understanding of the potential mechanisms of action related to effects of *H. erinaceus* on cognition and mood is currently limited and should be a focus of future research.

## Data Availability

The raw data supporting the conclusions of this article will be made available by the authors, without undue reservation.
